# A computer model of lens structure and function predicts experimental changes to steady state properties and circulating currents

**DOI:** 10.1186/1475-925X-12-85

**Published:** 2013-08-30

**Authors:** Ehsan Vaghefi, Nancy Liu, Paul J Donaldson

**Affiliations:** 1 Department of Optometry and Vision Sciences, University of Auckland, Building 502, Level 4, 85 Park Road, Grafton, Auckland, New Zealand; 2 Auckland Bioengineering Institute, University of Auckland, Auckland, New Zealand; 3 School of Medical Sciences, University of Auckland, Auckland, New Zealand

**Keywords:** Computational modelling, Ocular lens, Microcirculation, Finite element, Physiological perturbations

## Abstract

**Background:**

In a previous study (Vaghefi et al. 2012) we described a 3D computer model that used finite element modeling to capture the structure and function of the ocular lens. This model accurately predicted the steady state properties of the lens including the circulating ionic and fluid fluxes that are believed to underpin the lens internal microcirculation system. In the absence of a blood supply, this system brings nutrients to the core of the lens and removes waste products faster than would be achieved by passive diffusion alone. Here we test the predictive properties of our model by investigating whether it can accurately mimic the experimentally measured changes to lens steady-state properties induced by either depolarising the lens potential or reducing Na^+^ pump rate.

**Methods:**

To mimic experimental manipulations reported in the literature, the boundary conditions of the model were progressively altered and the model resolved for each new set of conditions. Depolarisation of lens potential was implemented by increasing the extracellular [K^+^], while inhibition of the Na^+^ pump was stimulated by utilising the inherent temperature sensitivity of the pump and changing the temperature at which the model was solved.

**Results:**

Our model correctly predicted that increasing extracellular [K^+^] depolarizes the lens potential, reducing and then reversing the magnitude of net current densities around the lens. While lowering the temperature reduced Na^+^ pump activity and caused a reduction in circulating current, it had a minimal effect on the lens potential, a result consistent with published experimental data.

**Conclusion:**

We have shown that our model is capable of accurately simulating the effects of two known experimental manipulations on lens steady-state properties. Our results suggest that the model will be a valuable predictive tool to support ongoing studies of lens structure and function.

## Background

**Figure 1:**
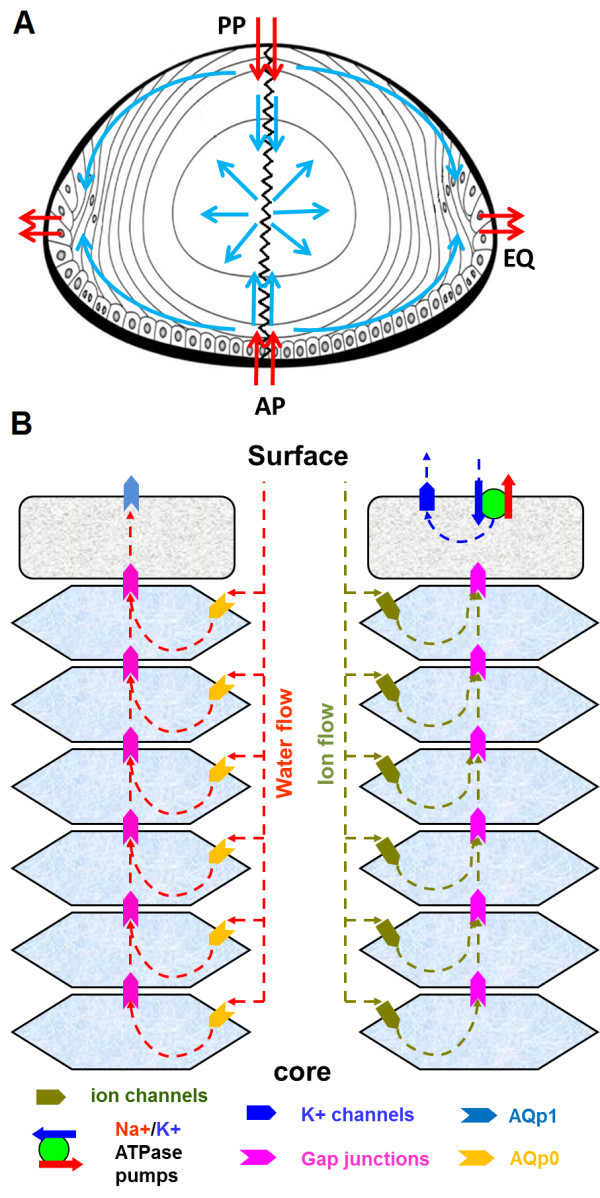
**Lens structure and function. A**: Schematic diagram of an axial view of the lens showing a model of solute penetration into the bovine lens. The anterior surface of the lens is covered by a single layer of epithelial cells which divide at the equator (EQ) to produce the elongating differentiating fiber cells. These cells eventually lose their nuclei and cellular organelles to become mature internalized fiber cells in the lens core. Fiber cells from adjacent hemispheres meet at the anterior (AP) and posterior (PP) poles to form the sutures. Arrows in the diagram represent the direction of ion and water fluxes. These fluxes have been directly measured outside the lens (red arrows) [2,15,16], but their position and direction inside the lens are to date purely theoretical (blue arrows) [1,17]. **B**: An equatorial cross section of the lens showing a cellular view of ion and water movement in the lens. Water and solutes are proposed to flow into the lens via the extracellular space, cross fiber cell membranes, and flow outward via an intracellular pathway mediated by gap junction channels.

In the absence of blood supply it appears that the ocular lens operates an internal microcirculation system [1,2]. This system ensures that the transparency and optical properties of the lens are maintained by delivering nutrients, removing wastes and preserving its ionic homeostasis [Figure 1A] [3,4]. This circulation is thought to be driven by a circulating flux of Na^+^ ions that enters the lens via the extracellular space between fiber cells, before eventually crossing fiber cell membranes, and then flowing from cell-to-cell towards the surface, via an intracellular pathway mediated by gap junction channels [Figure 1B] [1,5,6]. The gap junction coupling conductance in the outer shell of differentiating fibers is concentrated at the equator [7,8]. Hence, the intracellular current is directed towards the equatorial epithelial cells, where the highest densities of Na^+^/K^+^ pumps are located to actively transport Na^+^ out of the lens [9]. Thus, the intracellular current effluxes are highly concentrated at the equator, causing the measured net current flow in this region to be outward [10,11]. At the poles, there is very little intracellular current so the measured net current is predominantly inward, along the extracellular spaces [Figure 1B] [12,13]. The driving force for these fluxes is hypothesized to be the difference in the electromotive potential of surface cells that contain Na^+^/K^+^ pumps and K^+^-channels, and inner fiber cells which lack functional Na^+^/K^+^ pumps and K^+^-channels and whose permeability is dominated by non-selective cation and Cl^-^ conductances [14]. This electrical connection together with the different membrane properties of the surface and inner cells causes the standing current to flow. In this model, it is proposed the circulating currents measured at the lens surface drive a net flux of ions within the lens that in turn generates fluid flow. The extracellular flow of water in turn convects nutrients towards the deeper lying fiber cells, while the intracellular flow removes wastes and creates a well-stirred intracellular compartment [Figure 1A] [14].

While the experimental evidence in favour of this model is accumulating [5], [11,15,18-29] it is still somewhat controversial [30,31]. In an attempt to improve our understanding of lens structure and function we have developed a 3D computer model that utilises finite element modelling (FEM) to encapsulate structural features of the lens such as fiber cell orientation, extracellular space dimensions and gap junction distribution, plus functional information on the spatial differences in membrane permeability between the surface and inner lens cells that are thought to drive the circulating currents [17,26,28]. Using a series of experimentally derived boundary conditions [32-34] to solve the model, we showed that our model is not only capable of accurately predicting experimentally measured steady state lens properties, but also generates circulating ion and water fluxes as predicted by the microcirculation model [17,26].

In this paper, we report on further testing of our computer model of lens structure and function, and show that it is capable of predicting experimentally measured changes in the lens steady state properties and circulating fluxes, induced by either membrane depolarization or inhibition of the Na^+^ pumps [2,15,16,19,33]. We have further complemented our modelling approach by performing a series of experimental measurements of the effect of elevated extracellular K^+^ on lens voltage. The ability of our model to accurately predict the effects of published experimental perturbations on lens function shows that our model has the potential to offer insights into how changes in lens physiology can lead to changes in lens transparency and ultimately cataract.

## Methods

### Computer model

The expansion of the equations that govern ion and fluid dynamics in the lens [2,5,14,35-38] to 3D, and their subsequent implementation into a finite element mesh that encapsulates the known structural and functional parameters of the mouse lens has been fully described in a previous publication [17]. The assumptions and a summary of the major equations used in formulating the model are briefly listed below.

#### Fluid fluxes

The Stokes equations, a simplified version of the Navier–Stokes equations, which are derived from the conservation of mass, momentum, and energy [39], were used to model lens fluid fluxes. To simplify the non-linear Navier–Stokes equations to Stokes equations, it was assumed that water in the lens is an incompressible Newtonian fluid with a spatially constant viscosity at steady state; such that it can be described as a “creeping” (low-Reynolds number) flow with ignorable turbulence [40]. Using these assumptions the general Navier–Stokes equations were simplified to the following linear equations [Equation 1, Equation 2] the parameters and units of which are listed in Table 1.

(1) ∇.u=0

(2) $$-\nabla p+\mu \nabla^{2}u+\mathrm{pf}=0$$

**Table 1:** Glossary of symbols used in this manuscript

**Symbol**	**Description**	**Units**
***C***	Concentration	mM
***D***	diffusion coefficient	m^2^/s
***e***	electron charge	C
***j***	fluid flux	mol/(m^2^s)
***k***_***B***_	Boltzmann constant	J/K
***p***	Pressure	Pas
***T***	Temperature	K
***f***	body force	N/Kg
***z***	Valence	-
***α***	solute species	-
***μ***	dynamic viscosity	N.s/m^2^
***ρ***	mass density	Kg/m^3^
***φ***	Potential	V
***L***_***p***_	intercellular hydraulic permeability	m^3^/(N.s)
***Os***	Osmolarity	Osm/L
***σ***	membrane reflectance	-
***g***	conductivity per membrane area	S/m^2^
***F***	Faraday constant	C/mol
***u***	Velocity	m/s

The above equations were used to calculate the extracellular, trans-membrane and intracellular fluid fluxes that described the flow of water across fibre cell membrane between the extracellular and the intracellular spaces. To represent these fluxes the fibre cell membrane was considered as a semi-permeable membrane [26,38] through which fluid passed due to a combination of hydrostatic and osmotic pressure gradients [41]. We used the following equation to calculate the velocity of the trans-membrane water fluxes [26,38,41].

(3) $$u_{m}=-L_{p}\Delta p-\sigma L_{P}\mathrm{RT}\Delta \mathrm{Os}$$

The parameters and their units are listed in [Table 1].

#### Ion fluxes

Ionic fluxes in the lens are governed by diffusion, electro-diffusion and advection and were modelled using the Nernst-Plank equation with an added advection term [Equation 4, Table 1] [28,38].

(4) ja=−Da∇Ca−zaeDakBT∇φ.Ca+Ca

This equation was used to model the ionic intracellular and extracellular fluxes which were linked by implementation of a trans-membrane flux [1,26,38] described by the following equations [Equation 5–7].

(5) $$j_{a}=\frac{g_{a}}{F}\left(V_{m}-E_{a}\right)$$

(6) $$E_{a}=-\frac{k_{B}T}{z_{a}e}\ln\left(\frac{C_{a2}}{C_{a1}}\right)$$

(7) $$V_{m}=\varphi_{i}-\varphi_{e}$$

The parameters and their units are listed in [Table 1]. In the above equations, *E* is the Nernst potential. The modelled ions (i.e. Na^+^, K^+^ and Cl^-^) accompanied the trans-membrane water fluxes into the cells. The membrane conductivity for each modelled ion had been calculated based on experimental data [5,31,35,42] which we used for various modelled trans-membrane ion fluxes.

#### Finite element mesh creation

**Figure 2:**
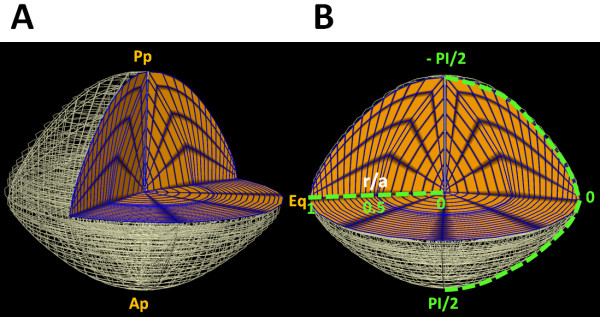
**Visualization of modelling results. A**: 3D quarter section view of finite element mesh on which the model is solved. Points of reference are labelled on these diagrams, (Ap) Anterior pole, (Pp) Posterior pole and (Eq) the equator. **B**: Rotated view of (A) with the quarter-view facing out to illustrate how 2D plots of radial voltage and concentration gradients and circumferential surface plots of current density were extracted from the 3D model. Values for voltage and concentration were plotted against normalised lens radius (r/a) where 0 indicates the core and 1 the lens periphery. Surface currents were plotted against the angle from the equator, with the equator defined as 0, the posterior and anterior poles were –PI/2 and PI/2 radians, respectively.

All water and ion flux equations were implemented on a representative finite element mesh constructed of the mouse lens to create an interlinked system of equations that could be solved using a set of boundary conditions that represented the ionic concentrations at the lens surface [Table 2]. An anatomically accurate scaffold of an adult mouse lens with an equatorial radius of 0.125 cm, a posterior thickness of 0.1 cm and anterior thickness of 0.085 cm was created to implement our modelling approach [Figure 2A] [43]. A cylindrical polar coordinate system (r, θ, z) and Cubic Hermite basis function were used to create a smooth 3D computational mesh of the mouse lens. The computer meshing algorithm put an ellipsoid volume (representing the outer regions of the mice lens) on the top of a spherical centre (representing its core). In our other in-vitro experiments, we have observed that the nucleus of the lens is almost completely spherical, while the outer layers add to the final elliptical shape of the lens. In our model, the transition between the spherical core and elliptical outer region happened at the r/a = 0.5.

**Table 2:** Initial conditions at outer lens boundary for the present model, under “normal” conditions

**Species**	**Description**	**Quantity**	**Units**
**Na**_**eo**_	Extracellular sodium concentration	110	mM
**K**_**eo**_	Extracellular potassium concentration	8	mM
**Cl**_**eo**_	Extracellular chloride concentration	115	mM
**Na**_**io**_	Intracellular sodium concentration	7	mM
**K**_**io**_	Intracellular potassium concentration	100	mM
**Cl**_**io**_	Intracellular chloride concentration	10	mM
**T**	Temperature	310	K

A bi-domain modelling approach [38,44,45] was adopted in which every element in the mesh represented a cluster of many fiber cells and enclosed extracellular space to reflect the relationship between the intra- and extra-cellular spaces. Solute and fluid flow equations were coupled in the FEM mesh using the C++ programming language. The model was solved using an experimentally derived set of starting boundary conditions [Table 2]. To mimic depolarization of the lens potential, or reduction Na^+^ pump activity these starting boundary conditions were altered as described in the text; and the model resolved for each set of novel conditions.

#### Model solution

An adaptive and iterative Euler method capable of achieving a converged steady state solution [38] was used to solve the model for each of the different boundary conditions. Each iteration of the adaptive Euler method utilised several steps to solve the coupled solute and fluid transport equations, followed by a solution update step as described previously [17,28,38]. The model was stable under the simulated conditions and converged on a singular set of answers. An in-house graphical interface, utilizing text formatted files of different fields, linked via JAVA programming language format to CMGUI (http://www.cmgui.org), was used to create a 3D representation of the model, from which quarter views were extracted for visualisation [Figure 2]. In order to facilitate comparison of the calculated parameters with those generated experimentally, field values across the equatorial radius of the lens were extracted from the 3D model and plotted against normalized radial distance (r/a), in order to generate conventional 2D plots [Figure 2B]. Furthermore, fields such as net current densities (*I*_*net*_) are visualized here on the surface of the computer mesh. For those visualizations we have used plots across the outer boundary of the mesh, marked on [Figure 2B], where the posterior pole is at *–PI/2*, anterior pole is *+ PI/2* and equator is assigned to *0* radians.

### Experimental measurements of lens potential

To obtain an experimental data set in the rat lens that could be compared to our model, microelectrode measurements were performed in extracellular solutions of varying K^+^ concentration.

#### Animals

All animals used in this study were treated in accordance with institutional guidelines and the ARVO Resolution on the Use of Animals in Research. All chemicals were obtained from Sigma (Sigma Chemical Company, St. Louis, MO) unless stated otherwise. Wistar rats 3–4 weeks of age were sacrificed by CO_2_ asphyxiation and cervical dislocation using protocols approved by the University of Auckland Animal Ethics Committee (AEC R188). Eyes were extracted and the lenses were then dissected and placed in temperature controlled Artificial Aqueous Humour (AAH: 124 mM NaCl, 0.5 mM MgCl_2_, 4 mM KCl, 10 mM NaHCO_3_, 2 mM CaCl_2_, 5 mM glucose, 10 mM HEPES and 20 mM sucrose, pH 7.4, 300 mOsM.kg^-1^).

#### Membrane potential measurements

The lenses were placed in recording chamber on the stage of a dissecting microscope and continually perfused with warm AAH. The resting potential of the lens (*E*_*m*_) was recorded by impaling the lens with a microelectrodes connected to the head-stage of a microelectrode amplifier (Axoclamp-2A, Axon instruments, Union City, CA). The output from the amplifier was digitized (DigiData 1200, Axon Instruments), and acquired (AxoScope, Axon Instruments) before being analysed off-line (Clampfit, Axon Instruments). To monitor the effect of changing the extracellular K^+^ concentration on *E*_*m*_, the bath was then perfused with a range of concentrations of AAH ringers in which the NaCl was replaced with an equimolar concentration of KCl. All equipment was grounded and placed inside a Faraday cage to minimise electrical interference.

## Results

Previously, we have solved our computational model using a set of boundary conditions [Table 2] that represents the “normal” ionic environment experienced by the lens in vivo [17]. Using these conditions, we produced 3D maps of standing fields of intracellular and extracellular ion concentrations, electrical potentials and circulating ionic and water fluxes that agreed with the published literature [14,36,46,47]. Here we have altered the boundary conditions to firstly mimic lens depolarisation, and then a reduction in Na^+^ pump rate. These two perturbations are known to affect the direction and magnitude of circulating currents measured at the lens surface [9,15,34,48,49]. By resolving the model under these new boundary conditions and comparing the data generated for electrical potentials, and the net circulating currents with our own and existing experimental data, we were able to assess the ability of our model to predict how changes in the underlying physiology of the lens affect its circulation system. Finally, we have used the model to predict changes to standing fields of intracellular ionic concentrations induced by these two perturbations.

### Depolarization of the lens potential by high extracellular K^+^

Although epithelial (E_K_ ~ −80 mV) and deeper fiber cells (E_NSC_ ~ 0 mV) have distinctly different resting membrane potentials, the fact that they are extensively coupled by gap junctions [14,50,51] means that the lens potential (*E*_*m*_), as measured by an intracellular microelectrode, represents the weighted average of all cells. Microelectrode measurements of *E*_*m*_ from a variety of species of lens have shown that the potential is around ~ −70 mV in magnitude [5,52,53] indicating that it is dominated by the K^+^ conductance localised to epithelial and peripheral differentiating fiber cells. If however, the microelectrode is incrementally advanced into the lens, the measured potential decreases slightly to ~ −50 mV [46]. This indicates that a standing gradient in electrical potential exists in the lens. Increasing the concentration of extracellular K^+^ lens bathing medium reduces E_K_ in these surface cells, causing an overall depolarization of the lens potential [33], a flattening of the electrical gradient and a reduction in magnitude of ion currents recorded at the lens surface [9,15,16].

**Figure 3:**
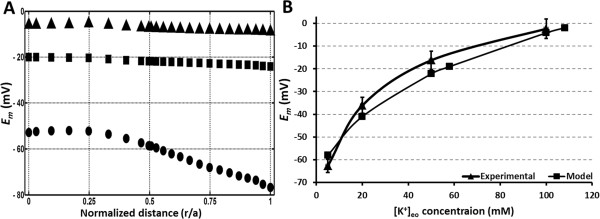
**Effect of increasing extracellular K + on lens electrical potential. A**: Equatorial plots of lens voltage (Em) versus radial distance (r/a) showing the electrical potential gradients extracted from the model for three different extracellular K + concentrations (● = 8; ■ = 58; and ▲ = 108 mM [K+]eo). **B**: Plot showing the effect of changing the extracellular K + concentration ([K+]eo) on the lens potential measured experimentally by microelectrodes located in the outer cortex (▲) and values obtained from the model (■) as an average of the equatorial electrical gradients shown in **A**.

To test whether our model could predict similar changes to the lens electrical gradient, we gradually changed the boundary conditions used to solve the model by increasing [K^+^]_eo_ and decreasing [Na^+^]_eo_ in 10 mM steps from the “normal” concentrations of [K^+^]_eo_ = 8 and [Na^+^]_eo_ = 110. This approach ensured that the total cation (Na^+^ and K^+^) content remained constant, while preserving the other initial boundary conditions [Table 2]. Here we show model predictions of *E*_*m*_ versus distance into the lens (r/a), for three selected sets of cation concentrations ([K^+^]_eo_ = 8, [Na^+^]_eo_ = 110; [K^+^]_eo_ = 58, [Na^+^]_eo_ = 57; and [K^+^]_eo_ = 108, [Na^+^]_eo_ = 10 mM) used as starting boundary conditions [Figure 3A].

Using the “normal” cation concentrations ([K^+^]_eo_ = 8, [Na^+^]_eo_ = 110) the model generates a standing electrical gradient that is -76 mV at the periphery and declines to -52 mV in the lens core [Figure 3A]. Changing the cation concentrations to [K^+^]_eo_ = 58, [Na^+^]_eo_ = 60 mM and [K^+^]_eo_ = 108, [Na^+^]_eo_ = 10 mM, caused a progressive depolarization of *E*_*m*_ towards 0 mV and abolished the radial gradient in electrical field as would be expected if the *E*_*m*_ of the lens is dominated by E_K_ [Figure 3A].

We then experimentally validated our computer model’s predictions by performing microelectrode measurements of *E*_*m*_ in rat lenses exposed to changes in the extracellular cation concentration [Figure 3B]. In these experiments *E*_*m*_ values were collected by microelectrodes that were located in the outer cortex of lens. To facilitate comparison between the experimental data collected from a single location and our calculated electrical gradients, we averaged the electrical gradients obtained from the computational model to generate a single value of *E*_*m*_ for the different sets of cation concentrations [Figure 3B]. It was apparent from these comparisons that the model lens is depolarized when its ionic balance is disturbed.

**Figure 4:**
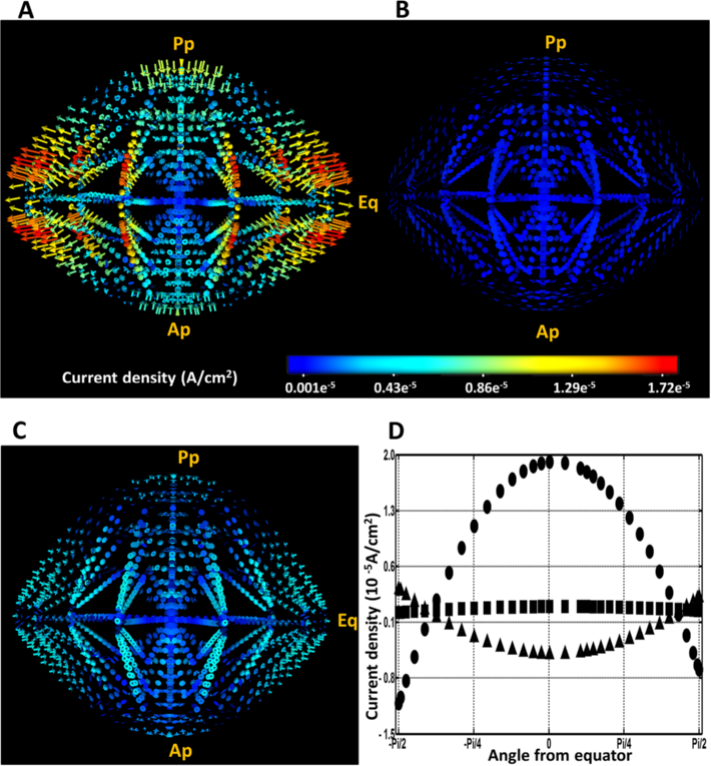
**Effect of lens depolarisation on net current density.** 3D representation of the predicted net current density patterns obtained for solving the model using [K+]eo concentrations of **(A)** 8 mM, **(B)** 58 mM, and **(C)** 108 mM. The numbers on the colour-bar are in A/cm^2^ units. **D**: 2D profiles of the change in current density at the surface of the lens going in a circumferential direction from posterior pole (−Pi/2) to anterior pole (Pi/2) expressed as a function of the angle form the equator (0) extracted from the 3D plots **(A-C)** created by solving model using [K+]eo concentrations of 8 mM (●), 58 mM (■), and 108 mM (▲).

Such decline of trans-membrane potentials was expected to have an impact on the ionic circulatory fluxes. Indeed vibrating probe [15,16] and modified Ussing chamber [9] experiments have shown that net current densities are directed inwards at the poles and outwards at the equator. In a previous study we used our computer model to visualise net current flows through the lens in 3D [26] [Figure 4A]. Furthermore, we demonstrated that our model’s predictions agreed in net magnitude and direction with the experimentally measured currents around the surface of the lens [17]. In this study we have investigated the effects lens depolarization induced by elevating extracellular potassium ([K^+^]_eo_) on these calculated current densities.

From the 3D current density (*I*_*net*_) maps calculated by the model, it was apparent that depolarisation of *E*_*m*_, caused by increasing [K^+^]_eo_ from 8 to 58 mM, resulted in a substantial reduction in inwardly and outwardly directed currents at the poles and equator, respectively [Figure 4B]. Interestingly, a further increase in [K^+^]_eo_ to 108 mM and depolarization of *E*_*m*_ to ~ 0 mV caused the calculated *I*_*net*_ vectors to reverse. These vectors appeared to become inward at the equator and outward at the polar regions [Figure 4C]. To facilitate comparison between the different conditions, we extracted the 2D magnitude plots of the calculated surface *I*_*net*_ field and plotted them against the angle from the equator [Figure 2B]. This analysis more clearly highlights the reduction in calculated surface current densities, caused by increasing [K^+^]_eo_ to 58 mM and the reversal of the predicted *I*_*net*_ field by increasing [K^+^]_eo_ to 108 mM [Figure 4D].

The effects of increasing [K^+^]_eo_ on the predicted magnitude and directionality of surface current densities in our model of the mouse lens were found to be in agreement with experimental findings for a variety larger lenses obtained from rats [16], frogs [15] and rabbits [9] [Table 3]. Although comparing the results from different species can be problematic since the absolute magnitudes of circulating surface currents recorded are different and the measurement techniques vary, all lenses responded in a similar fashion to the replacement of extracellular Na^+^ with K^+^. Partial replacement of Na^+^ with K^+^ decreased the magnitude of circulating currents in all the lenses and full replacement eventually caused the direction current flow to reverse in all lenses.

**Table 3:** Comparison between the computational model of the mouse lens and experimental data collected from different species

**Species**	**Technique**	**Medium cation concentrations (mM)**	Current values* and % change from control		
			**Ap**	**Eq**	**Pp**
**Mouse**^**§**^	Computational modelling	[K^+^]_eo_ = 8 - [Na^+^]_eo_ = 110	- 8.5 μA/cm^2^	+ 20 μA/cm^2^	- 11 μA/cm^2^
		[K^+^]_eo_ = 58 - [Na^+^]_eo_ = 57	85%	90%	85%
		[K^+^]_eo_ = 108 - [Na^+^]_eo_ = 10	- 35%	- 25%	- 40%
**Rat **[16]	Vibrating probe	[K^+^]_eo_ = 5 - [Na^+^]_eo_ = 130	- 20 μA/cm^2^	+ 22 μA/cm^2^	- 12 μA/cm^2^
		[K^+^]_eo_ = 75 - [Na^+^]_eo_ = 75	N/A	- 75%	N/A
		[K^+^]_eo_ = 113 - [Na^+^]_eo_ = 37	N/A	- 200%	N/A
**Frog **[15]	Vibrating probe	[K^+^]_eo_ = 2 - [Na^+^]_eo_ = 113	- 13 μA/cm^2^	+ 24 μA/cm^2^	- 36 μA/cm^2^
		[K^+^]_eo_ = 54 - [Na^+^]_eo_ = 54	60%	60%	60%
		[K^+^]_eo_ = 105 - [Na^+^]_eo_ = 2.5	- 70%	- 70%	- 60%
**Rabbit **[9]	Ussing chamber	[K^+^]_eo_ = 3 - [Na^+^]_eo_ = 115	- 1.2 μA	+ 10.8 μA	- 2.9 μA
		[K^+^]_eo_ = 37 - [Na^+^]_eo_ = 83	- 50%	N/A	40%

^§^Mouse data is from the current model. ^*^Current values obtained from the model and the literature are expressed as either current densities (μA/cm^2^) or magnitudes (μA).

While the agreement of the simulated and the measured trends is encouraging, the variance of quoted absolute values indicate that our model should be optimised for each species of lens to accurately predicting the physiological state in a specific species. In summary, our model has confirmed that differences in membrane permeability’s determine the magnitude and directionality of circulating currents in the lens.

### Na^+^/K^+^ ATPase pumps’ rate reduction

It is been proposed that the active removal of Na^+^ ions at the lens surface is the major driver of the lens circulation system [23]. In this regard it has been shown that Na^+^/K^+^ ATPase activity in a variety of lenses is concentrated around the equatorial plane [12,54,55] and that pharmacological inhibition of the these pumps abolishes current outflow at the equator and inflow at the poles [5,9]. Na^+^ pumps are known to be temperature sensitive [48,56-58], and it has been shown that cooling the lens slows the pump rate to produce a reversible shift in cation concentrations that is manifested as an accumulation of intracellular Na^+^ and a depletion of intracellular K^+^[57]. In our model this temperature sensitivity of the Na^+^ pump is captured by [Equation 8].

(8) $$I_{P\left(T\right)}=I_{P\left(T=310K\right)}Q_{10}^{\left(T-310\right)/10}$$

Where *I*_*p(T)*_ is the Na^+^ pump’s rate *T* Kelvin degrees, *I*_*p(T=310K)*_ is the Na^+^/K^+^ ATPase pump rate’s at 310 Kelvin both of which are measured in A/cm^2^; and Q_10_ is the temperature coefficient for ionic transport by the Na^+^ pump. Since Q_10_ has been estimated in a variety of lens studies to be between 1.8 and 2 [48,58,59], we chose a value of 1.9 for Q_10_. To affect a change in pump rate we simply resolved the model over a range of temperatures (*T* = 310° to 280°K in 5°K increments), while maintaining the other boundary conditions constant [Table 2].

**Figure 5:**
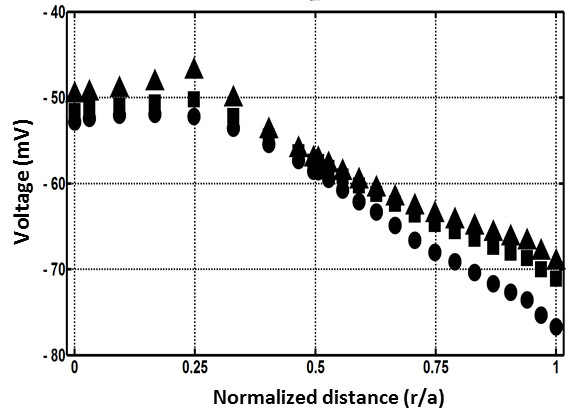
**Effect of reducing Na + pump rate on lens electrical potential. A**: Equatorial plots of lens voltage (Em) versus radial distance (r/a) showing the electric potential gradients extracted from the model for three different temperatures (● = 310; ■ = 300; and ▲ = 280°K) that reduce Na + pump rate.

Hence, we were able to use the inherent temperature variable (*T*) in our model to selectively reduce the rate of the Na^+^ pump. Such control enabled us to determine what effect pump rate has on electrical potential gradients and net surface current densities. Unlike the effects of increasing extracellular K^+^ [Figure 3], reducing the Na^+^ pump rate by either 77% (*T* = 300°K) or 90% (*T* = 280°K) produced only a small depolarisation of *E*_*m*_, and did not abolish the standing electrical gradient [Figure 5]. This differential effect of the two perturbations on the *E*_*m*_ calculated by our model supports the findings from experiments that *E*_*m*_ is primarily determined by the E_K_ of surface cells and indicates that any direct contribution from the electro-genic Na^+^ pump to the lens potential is minimal.

**Figure 6:**
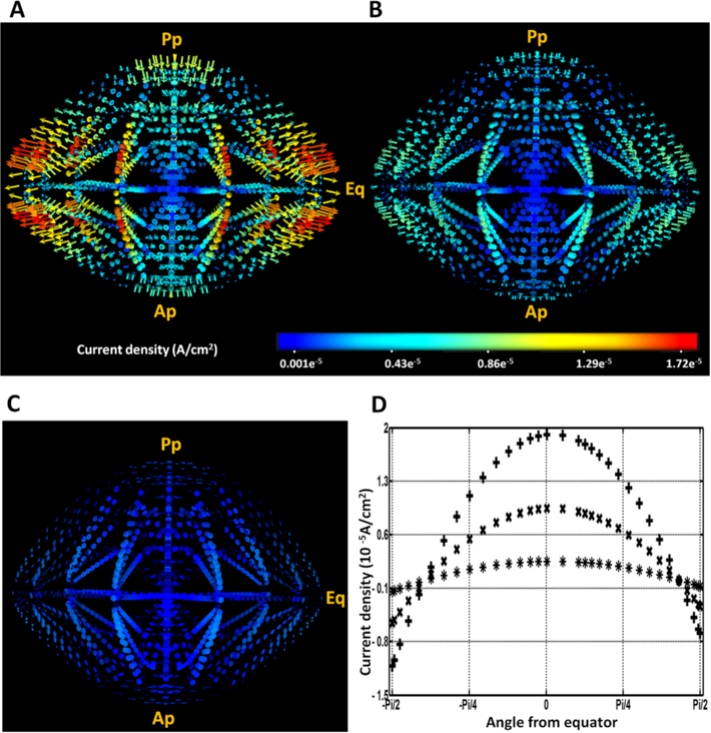
**Effect of reducing Na + pump rate on net current density.** 3D representation of the predicted net current density patterns obtained for solving the model using temperatures of **(A)** 310, **(B)** 300, and **(C)** 280°K as boundary conditions. The numbers on the colour-bar are in A/cm2 units. **D**: 2D profiles of the change in current density at the surface of the lens going in a circumferential direction from posterior pole (−Pi/2) to anterior pole (Pi/2) expressed as a function of the angle form the equator (0) extracted from the 3D plots **(A**-**C)** created by solving the model using temperatures of 310 (●), 300 (■), and 280°K (▲).

In contrast to the observed minimal effect on electrical gradients, reducing the activity of the Na^+^ pump had a major effect on the calculated 3D *I*_*net*_ vector fields [Figure 6A-C]. To highlight these changes, surface plots of *I*_*net*_ were extracted from the 3D vector fields [Figure 2B] for the different temperatures and are compared in Figure 6D. From this comparison it was apparent that reducing the Na^+^ pump rate, by lowering the temperature to 300 or 280°K, decreases the maximum calculated *I*_*net*_ by ~ 42% and 83%, respectively. This outcome demonstrates that the Na^+^ pumps are the major driver of the circulating currents in the lens; a result that is consistent with experimental findings in the literature. Lowering the temperature of the media bathing lenses in vitro to just above freezing point has also been shown to reduce ionic transport by 85 to 90% [56-58]. Furthermore, the pharmacological inhibition of the Na^+^ pumps in the lens with ouabain, [9,11,16,56] produced a dose dependent reduction of current densities at the surface of different species of lens [Table 4].

**Table 4:** **Comparison of inhibiting Na**^**+**^**/K**^**+ **^**pump rate either computationally or experimentally using Ouabain**

**Species**	**Temperature dependent % reduction in pump rate**	**% Reduction current density**		
		**Ap**	**Eq**	**Pp**
Mouse	28% (T = 300°K)	50%	50%	50%
Model	82% (T = 280°K)	100%	100%	100%
	**Pharmacological inhibition of pump rate Ouabain (mM)**			
Frog [16]	0.1 mM	50%	70%	40%
Rabbit [15]	0.1 mM	60%	60%	60%
Rat [9]	1 mM	100%	100%	100%

In summary, it appears that our mouse model is in general agreement with experimentally obtained measurements of *I*_*net*_ ion a variety of species, perturbed by elevated [K^+^]_eo_ or reduced Na^+^ pump rate.

To further investigate the effects of these two perturbations on the underlying lens physiology, we examined the ability of our model to predict changes in intracellular concentration gradients in response to lens depolarization and a reduction in Na^+^ pump rate.

### Calculated intracellular ion concentration gradients

**Figure 7:**
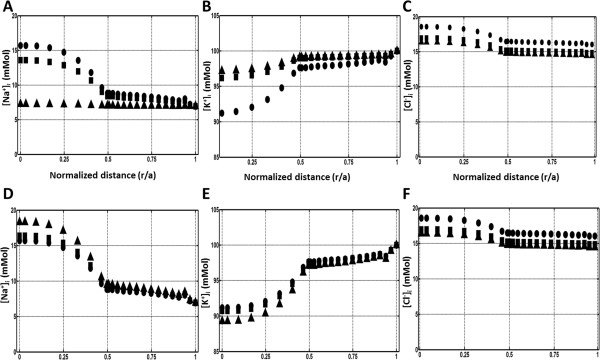
**Comparison of the effects of depolarization and reduction in Na + pump rate on intracellular ion concentration gradients. A**-**C**: 2D plots of the radially extracted intracellular concentration gradients for Na + **(A)**, K + **(B)** and Cl- **(C)** obtained by solving the model using extracellular K + concentrations of 8 (●), 58 (■) and 108 mM (▲) as the boundary conditions to mimic lens E_m_ depolarization. D-F: 2D plots of the radially extracted intracellular concentration gradients for Na + **(D)**, K + **(E)** and Cl- **(F)** obtained by solving the model using temperatures of 310 (●), 300 (■) and 280°K (▲) as the starting boundary conditions to mimic a reduction in Na + pump rate.

It has been shown experimentally that a radial concentration gradient exists for Na^+^ in the mouse lens where Na^+^ is lowest (~7 mM) in peripheral fiber cells and highest (~16 mM) in the lens nucleus [16,60]. The existence of this gradient is intuitively expected based on the distributed passive Na^+^ permeability that drives the entry of Na^+^ ions into all fiber cells and the localised expression of Na^+^ pumps to peripheral cells that mediates the active removal of Na^+^ from the lens. Our model was able to reproduce this measured gradient in intracellular [Na^+^] [Figure 7A&D], but our data appear to contain a discontinuity at r/a = 0.5 which was not observed in the fit to the experimental data. This slight discrepancy between the shape of the measured and calculated gradients could reflect either the smoothing effect of fitting a trend curve the inherent scatter of the experimental data [16,60] or to a potential caused by the transition of the mesh from an ellipsoid to a spherical representation in the cortex and core, respectively [Figure 2]. The model also calculated the steady state standing gradients for [K^+^] and [Cl^-^] that have yet to be measured experimentally. The model predicts a gradient for [K^+^]_i_ [Figure 7B&E] that is opposite to that found for [Na^+^]_i_, with [K^+^] being lowest in the core and highest in the periphery, so that the total cation content is balanced in the different regions of the lens. In contrast, [Cl^-^]_i_ was estimated to be relatively constant throughout the lens [Figure 7C&F], a prediction consistent with the role of [Cl^-^] in maintaining the electro-neutrality of the lens.

Interestingly, depolarizing the lens *E*_*m*_ [Figure 7A-C], or reducing Na^+^ pump rate [Figure 7D-F], had different effects on the modelled intracellular ion concentration gradients. Depolarizing the lens by progressively increasing [K^+^]_eo_ to 108 mM produced a flattening of the [Na^+^]_i_ gradient [Figure 7A], while reducing pump rate by lowering the temperature only slightly elevated this gradient [Figure 7D]. We observed a similar, but in the opposite direction, effect of these two perturbations on the [K^+^]_i_ gradient [Figure 7B&E], but only minor changes in modelled [Cl^-^]_i_ gradient [Figure 7C&F]. Our modelling predictions are similar to the “cation shift” effect mentioned in the literature [57]. At low temperatures, an accumulation of [Na^+^]_i_ and depletion of [K^+^]_i_ in the lens has been experimentally observed [57], [48]. It has also been shown that this “cation shift” effect was reversible, by restoring the temperature to 37°C. In our model decreasing the temperature decreased the [K^+^]_i_ concentration in the core of the lens by around 4 mM, meanwhile the [Na^+^]_i_ concentration was raised by the same amount in the centre of the model [Figure 7D-F]. So in effect our model can predict the “cation shift” phenomenon.

## Limitations of the model

It worth noting that any computational model, including the one presented here, is at best an approximation of a complex biological tissue. All such models require incremental improvement as new experimental information becomes available and improved methods for solving the model are developed. For example, our current model estimates irregular ionic concentration gradients [Figure 7], which is not consistent with the measured smooth curves of these profiles throughout the lens [16]. We believe that these estimations will be improved in future upgrades of our model utilizing higher resolution finite element meshes to capture the fine 3D geometry of the lens and finer regional distribution of elements such as the gap junctions and extracellular space tortuosity, all without compromising the computational load.

Furthermore, the under-estimation of the magnitude of hydrostatic pressure in the core (19.5 kPa versus 43 kPa) noted in our original study [30], is another example where our model deviates from recent experimental findings [16,60]. It has been proposed that the experimentally measured pressure gradient is generated by the restricted flow of water from the centre to the periphery of the lens through gap junction channels [1,14]. This illustrates that structural components of the lens can influence the magnitude of the pressure gradient and suggests that the difference between calculated and measured pressure fields may reflect the absence of a structural feature not currently captured in our model [61,62]. In this regard we have recently identified a zone in the inner cortex of the lens that exhibits a reduction in the penetration of solutes and water [25] that could influence the magnitude of the calculated pressure gradient. What is reassuring, however, is that our model correctly predicted the experimentally measured percentage change in hydrostatic pressure induced by either depolarising the lens or inhibiting pump rate. Experimental elevation of extracellular K^+^ decreased the measured hydrostatic pressure in the core by 90% [16], while our model calculated a similar change of 85%. Similarly inhibiting the Na^+^/K^+^ pump activity with ouabain produced a 50% drop in pressure [16], while a computationally induced reduction in pump rate of 82% produced a 75% drop in pressure. This qualitative association between the electrical gradients and pump rate activity suggests that circulating current and fluid fluxes are involved in generating the hydrostatic pressure gradient. The ability to quantitatively predict the magnitude of changes in the hydrostatic pressure gradient is an obvious area where further work will improve the accuracy of our model.

## Conclusions

In this paper we have tested the ability of our 3D computer model of lens structure and function to predict changes in the electrical field, net current densities, and intracellular ionic concentration gradients in response to a depolarization lens potential or a reduction in Na^+^ pump activity induced by an elevation in extracellular [K^+^] and lowering the temperature, respectively. The ability of our model to predict the effect of these perturbations on lens properties showed good agreement with the experimental data available in the literature for a variety of species of lens [9,11,15,16,48,56-58], thereby confirming that spatial differences in membrane permeability and Na^+^ pump rate are the major drivers of circulating currents in the lens.

While our current finite element model is based on a mouse lens, we believe that the microcirculation equations, derived in the literature [2,5,14] and implemented here, are applicable to other species. However, it should be noted that larger mammalian lenses, such as the human lens, have more complex structures compared to rodent lenses [43,61,62]. Capturing such complex structural features using an appropriate finite element mesh that is specific to each species of lens will enable us to model lens structure and function in different animal models and ultimately the human lens of different ages.

This modelling approach will afford us the capability to computationally isolate different components of the lens microcirculation system and study their effects on overall lens homeostasis. This ability to create “digital knockout” models of the ocular lens will facilitate our ability to design and analyse experiments in order to study the contribution of individual ion channels and transporters to the generation of the lens internal microcirculation system.

## Abbreviations

AP: Anterior pole; AAA: Artificial aqueous humour; EQ: Equator; FEM: Finite element modelling; Inet: Net current densities; PP: Posterior pole; r/a: Radial distance; Em: Resting potential; T: Temperature.

## Competing interests

The authors declare that they have no competing interests.

## Authors’ contribution

EV created and modified the computational model and drafted this manuscript. NL performed the microelectrode experiments. PD conceived the manuscript; final edited it and approved the final version. All authors read and approved the final manuscript.
